# Supplementary material for: Psychosocial Care for Adult Cancer Patients: Guidelines of the Italian Medical Oncology Association
Source: Cancers (Basel). 2021 Sep 29;13(19):4878. doi: 10.3390/cancers13194878 (PMC8508051; doi:10.3390/cancers13194878)
Supplement: Supplementary file 1 [file cancers-13-04878-s001.zip › cancers-1377410-supplementary.pdf]

Supplementary file: search strategies and study selection flow

Question 1: In patients with cancer, is information support provided by ward nursing staff indicated?

MEDLINE (PubMed) (1966 a December 2019)

((((((((("Neoplasms/psychology"[Mesh]) OR ((neoplasms[Title/Abstract] OR cancer[Title/Abstract] OR neoplasm*[Title/Abstract] OR tumo*[Title/Abstract]))))))) AND ((((((("Patient Education as Topic/methods"[Mesh:NoExp]) OR "Access to Information"[Mesh])) OR "Counseling/methods"[Mesh]) OR ("patient education"[Title/Abstract] OR "patient information"[Title/Abstract] OR "information needs"[Title/Abstract] OR "patient care planning"[Title/Abstract]))))))) AND ((((((("Teaching Materials"[Mesh]) OR ((audio* or video* or cassette* or tape or dvd* or compact dis* or cd or cds or multimedia or multimedia)))) OR (((("Internet"[Mesh]) OR "Telecommunications"[Mesh])) OR ((internet or web or website* or online or on line or electronic mail* or email* or mail* or blog* or weblog* or podcast* or portal* or computer program* or computer mediated or computer based or computer assisted)))) OR (((((telephon* or phone or phones or text messag* or sms))) OR ((pamphlet* or booklet* or leaflet* or flyer* or poster* or brochure* or print* material*))) OR ("education services" OR "information dissemination")))))

Records: 1001

Embase (Embase.com) (1974 a December 2019)

#1 'neoplasms psychology'/exp/mj
#2 neoplasms OR cancer OR neoplasm* OR tumo*:ti,ab
#3 #1 OR #2
#4 'patient education'/exp/mj
#5 'access to information'/exp/mj OR 'counseling'/exp/mj
#6 'patient education' OR 'patient information' OR 'information needs' OR 'patient care planning':ti,ab
#7 #4 OR #5 OR #6
#8 'teaching'/exp/mj
#9 'internet'/exp/mj OR 'telecommunication'/exp/mj
#10 (((((((((audio* OR video* OR cassette* OR tape OR dvd* OR compact) AND dis* OR cd OR cds OR multimedia OR internet OR web OR website* OR online OR on) AND line OR electronic) AND mail* OR email* OR mail* OR blog* OR weblog* OR podcast* OR portal* OR computer) AND program* OR computer) AND mediated OR computer) AND based OR computer) AND assisted OR telephon* OR phone OR phones OR text) AND messag* OR sms OR pamphlet* OR booklet* OR leaflet* OR flyer* OR poster* OR brochure* OR print*) AND material* OR 'education services' OR 'information dissemination':ti,ab
#11 #8 OR #9 OR #10
#12 #3 AND #7 AND #11
#13 'crossover procedure':de OR 'double-blind procedure':de OR 'randomized controlled trial':de OR 'single-blind procedure':de OR random*:de,ab,ti OR factorial*:de,ab,ti OR crossover*:de,ab,ti OR ((cross NEXT/1 over*):de,ab,ti) OR placebo*:de,ab,ti OR ((doubl* NEAR/1 blind*):de,ab,ti) OR ((singl* NEAR/1 blind*):de,ab,ti) OR assign*:de,ab,ti OR allocat*:de,ab,ti OR volunteer*:de,ab,ti

Records: 537

<u>2 records selected</u>

Question 2: Is it advisable to implement a service in the ward to ensure that all cancer patients who so wish can receive the information and related support they need?
Question 3: Does providing a list of possible questions to patients improve patient-physician communication?

MEDLINE (PubMed) (1966 a December 2019)

(Cancer OR neoplasm* Neoplasms"[Mesh]) AND (Physician-patient communication OR doctor-patient communication OR patient-physician communication OR patient-doctor communication OR Physician-Patient Relations[mh] OR "physician patient interaction" OR "doctor patient communication and interaction"

OR "communication skills") AND (intervention* OR decision aid* OR question prompt list* OR "question asking" OR question prompt sheet* OR tool* OR training)
Records: 1189

Embase (Embase.com) (1974 a December 2019)
#1 'neoplasms psychology'/exp/mj
#2 neoplasms OR cancer OR neoplasm* OR tumo*:ti,ab
#3 #1 OR #2
#4 'doctor patient relationship'/exp/mj OR 'physician-patient communication' OR 'doctor-patient communication' OR 'patient-physician communication' OR 'patient-doctor communication' OR 'physician patient interaction' OR 'doctor patient communication and interaction' OR 'communication skills':ti,ab
#5 intervention* OR decision OR aid* OR question OR 'prompt list' OR 'question asking' OR 'question prompt sheet' OR training:ti,ab
#6 #3 AND #4 AND #5
#7 'crossover procedure':de OR 'double-blind procedure':de OR 'randomized controlled trial':de OR 'single-blind procedure':de OR random*:de,ab,ti OR factorial*:de,ab,ti OR crossover*:de,ab,ti OR ((cross NEXT/1 over*):de,ab,ti) OR placebo*:de,ab,ti OR ((doubl* NEAR/1 blind*):de,ab,ti) OR ((singl* NEAR/1 blind*):de,ab,ti) OR assign*:de,ab,ti OR allocat*:de,ab,ti OR volunteer*:de,ab,ti
#8 #6 AND #7
Records:479

2 records selected

Question 4: Is the use of tools to favor patient involvement in decision-making at crucial time points of care indicated?

MEDLINE (PubMed) (1966 a December 2019)
(("Neoplasms/psychology"[Mesh] OR "neoplasms/complications"[Mesh] OR neoplasms[Title/Abstract] OR cancer[Title/Abstract] OR neoplasm*[Title/Abstract] OR tumo*[Title/Abstract]) AND "decision support techniques"[MeSH Terms] OR (‘Decision Support Technique’[Title/Abstract] OR ‘Decision Support Technics’[Title/Abstract] OR ‘Decision Support Technic’ [Title/Abstract] OR ‘Decision Support Model’[Title/Abstract] OR ‘Decision Support Models’[Title/Abstract] OR ‘Decision Modeling’[Title/Abstract] OR ‘Decision Aids’[Title/Abstract] OR ‘Decision Aid’[Title/Abstract] OR ‘Decision Analysis’ [Title/Abstract] OR ‘Decision Analyses’[Title/Abstract]) AND ((((((("Randomized Controlled Trial"[Publication Type]) OR "Clinical Trial"[Publication Type]) OR "drug therapy"[Subheading])) OR ((randomized[Title/Abstract] OR randomised[Title/Abstract] OR placebo[Title/Abstract] OR randomly[Title/Abstract] OR trial[Title/Abstract] OR groups)))) NOT ((("Animals"[Mesh]) NOT "Animals"[Mesh]) AND "Humans"[Mesh]))
Records: 2778

Embase (Embase.com) (1974 a December 2019)
#1 'neoplasms psychology'/exp/mj
#2 neoplasms OR cancer OR neoplasm* OR tumo*:ti,ab
#3 #1 OR #2
#4 'decision support system'/exp/mj
#5'decision support technique' OR 'decision support technics' OR 'decision support technic' OR 'decision support model' OR 'decision support models' OR 'decision modeling' OR 'decision aids' OR 'decision aid' OR 'decision analysis' OR 'decision analyses':ti,ab
#6 #4 OR #5
#7 'crossover procedure':de OR 'double-blind procedure':de OR 'randomized controlled trial':de OR 'single-blind procedure':de OR random*:de,ab,ti OR factorial*:de,ab,ti OR crossover*:de,ab,ti OR ((cross NEXT/1 over*):de,ab,ti) OR placebo*:de,ab,ti OR ((doubl* NEAR/1 blind*):de,ab,ti) OR ((singl* NEAR/1 blind*):de,ab,ti) OR assign*:de,ab,ti OR allocat*:de,ab,ti OR volunteer*:de,ab,ti
#8 #3 AND #6 AND #7
Records: 1104

2 records selected

Question 5: Can communication training addressed to patients favor participation and communication during the visit?

MEDLINE (PubMed) (1966 a December 2019)
((((((((("Neoplasms/psychology"[Mesh]) OR ((neoplasms[Title/Abstract] OR cancer[Title/Abstract] OR neoplasm*[Title/Abstract] OR tumo*[Title/Abstract]))))))) AND ((((((("Patient Education as Topic/methods"[Mesh:NoExp]) OR "Access to Information"[Mesh])) OR "Counseling/methods"[Mesh]) OR ("patient education"[Title/Abstract] OR "patient information"[Title/Abstract] OR "information needs"[Title/Abstract] OR "patient care planning"[Title/Abstract]))))))) AND ((((((("Teaching Materials"[Mesh]) OR ((audio* or video* or cassette* or tape or dvd* or compact dis* or cd or cds or multimedia or multimedia)))) OR (((("Internet"[Mesh]) OR "Telecommunications"[Mesh])) OR ((internet or web or website* or online or on line or electronic mail* or email* or mail* or blog* or weblog* or podcast* or portal* or computer program* or computer mediated or computer based or computer assisted)))) OR (((((telephon* or phone or phones or text messag* or sms))) OR ((pamphlet* or booklet* or leaflet* or flyer* or poster* or brochure* or print* material*))) OR ("education services" OR "information dissemination")))))
Records: 1001

Embase (Embase.com) (1974 a December 2019)

#1 'neoplasms psychology'/exp/mj
#2 neoplasms OR cancer OR neoplasm* OR tumo*:ti,ab
#3 #1 OR #2
#4 'patient education'/exp/mj
#5 'access to information'/exp/mj OR 'counseling'/exp/mj
#6 'patient education' OR 'patient information' OR 'information needs' OR 'patient care planning':ti,ab
#7 #4 OR #5 OR #6
#8 'teaching'/exp/mj
#9 'internet'/exp/mj OR 'telecommunication'/exp/mj
#10 (((((((((audio* OR video* OR cassette* OR tape OR dvd* OR compact) AND dis* OR cd OR cds OR multimedia OR internet OR web OR website* OR online OR on) AND line OR electronic) AND mail* OR email* OR mail* OR blog* OR weblog* OR podcast* OR portal* OR computer) AND program* OR computer) AND mediated OR computer) AND based OR computer) AND assisted OR telephon* OR phone OR phones OR text) AND messag* OR sms OR pamphlet* OR booklet* OR leaflet* OR flyer* OR poster* OR brochure* OR print*) AND material* OR 'education services' OR 'information dissemination':ti,ab
#11 #8 OR #9 OR #10
#12 #3 AND #7 AND #11
#13 'crossover procedure':de OR 'double-blind procedure':de OR 'randomized controlled trial':de OR 'single-blind procedure':de OR random*:de,ab,ti OR factorial*:de,ab,ti OR crossover*:de,ab,ti OR ((cross NEXT/1 over*):de,ab,ti) OR placebo*:de,ab,ti OR ((doubl* NEAR/1 blind*):de,ab,ti) OR ((singl* NEAR/1 blind*):de,ab,ti) OR assign*:de,ab,ti OR allocat*:de,ab,ti OR volunteer*:de,ab,ti
Records: 537

1 record selected

Question 6: Is communication skills training for healthcare professionals effective in improving healthcare professional communication outcomes?

MEDLINE (PubMed) (1966 a December 2019)
((((((((("Medical Oncology/education"[Mesh]) OR "Oncology Nursing"[Mesh])) OR ('medical oncology'[Title/Abstract] OR oncologist[Title/Abstract] OR nurse[Title/Abstract] OR 'oncology

nursing'[Title/Abstract]))) AND (((((("Education, Medical, Continuing"[Mesh]) OR "Education, Nursing, Continuing"[Mesh]) OR "Programmed Instructions as Topic"[Mesh])) OR ('communication skills'[Title/Abstract] OR training[Title/Abstract] OR education OR)) OR ('distance learning'[Title/Abstract] OR workshops[Title/Abstract]))) OR (‘Programmed Instruction as Topic’[Title/Abstract] OR ‘Self-Instruction Programs’[Title/Abstract] OR ‘Textbooks, Programmed’[Title/Abstract] OR ‘Programmed Textbook’ [Title/Abstract] OR ‘Programmed Learning’[Title/Abstract])))))))) AND (((((((((((("systematic review"[Title]) OR "meta-analysis "[Publication Type]) OR ("meta-analysis"[Title] OR "meta synthesis"[Title] OR "meta-analy*"[Title] OR "integrative review"[Title] OR "integrative research review"[Title])) OR "systematic literature review"[Title]) OR ("this systematic review"[Text Word] OR "pooling project"[Text Word]))) OR (("systematic review"[Title/Abstract]) AND review[Publication Type]))))))) OR ((((((((("Randomized Controlled Trial"[Publication Type]) OR "Clinical Trial"[Publication Type]) OR "drug therapy"[Subheading])) OR ((randomized[Title/Abstract] OR randomised[Title/Abstract] OR placebo[Title/Abstract] OR randomly[Title/Abstract] OR trial[Title/Abstract] OR groups)))) NOT ((("Animals"[Mesh]) NOT "Animals"[Mesh]) AND "Humans"[Mesh]))))
Records: 786

Embase (Embase.com) (1974 a December 2019)
#1 'oncology'/exp/mj OR 'oncology nursing'/exp/mj
#2 'medical oncology' OR oncologist OR nurse OR 'oncology nursing':ti,ab
#3 #1 OR #2
#4 'medical education'/exp/mj OR 'nursing education'/exp/mj OR 'teaching'/exp/mj
#5 'communication skills' OR training OR education OR 'distance learning' OR workshops OR 'programmed instruction as topic' OR 'self-instruction programs' OR 'textbooks, programmed' OR 'programmed textbook' OR 'programmed learning':ti,ab
#6 #4 OR #5
#7 #3 AND #6 AND ([cochrane review]/lim OR [systematic review]/lim OR [meta analysis]/lim)
Records: 1633

1 record selected

Question 7: Is the use of a screening intervention for distress indicated in patients with cancer?

MEDLINE (PubMed) (1966 a December 2019)
(((((((((("Neoplasms/psychology"[Mesh] OR "neoplasms/complications"[Mesh] OR neoplasms[Title/Abstract] OR cancer[Title/Abstract] OR neoplasm*[Title/Abstract] OR tumo*[Title/Abstract]))) AND (("Psychological Distress"[Mesh]) OR (distress[Title/Abstract] OR 'psychological distress'[Title/Abstract] OR 'emotional distress'[Title/Abstract])))) AND (("Early Detection of Cancer"[Mesh]) OR ("early Detection of Cancer"[Title/Abstract] OR test[Title/Abstract] OR screening OR)))) AND ((((((((("Randomized Controlled Trial"[Publication Type]) OR "Clinical Trial"[Publication Type]) OR "drug therapy"[Subheading])) OR ((randomized[Title/Abstract] OR randomised[Title/Abstract] OR placebo[Title/Abstract] OR randomly[Title/Abstract] OR trial[Title/Abstract] OR groups)))) NOT ((("Animals"[Mesh]) NOT "Animals"[Mesh]) AND "Humans"[Mesh]))))
Records: 1772

Embase (Embase.com) (1974 a December 2019)
#1 'neoplasms psychology'/exp/mj
#2 neoplasms OR cancer OR neoplasm* OR tumo*:ti,ab
#3 #1 OR #2
#4 ('early diagnosis' NEAR/5 screening) OR 'early detection' OR assess
#5 'depression'/exp/mj OR 'psychological distress' OR 'emotional distress' OR depression OR anxiety:ti,ab
#6 'crossover procedure':de OR 'double-blind procedure':de OR 'randomized controlled trial':de OR 'single-blind procedure':de OR random*:de,ab,ti OR factorial*:de,ab,ti OR crossover*:de,ab,ti OR ((cross NEXT/1 over*):de,ab,ti) OR placebo*:de,ab,ti OR ((doubl* NEAR/1 blind*):de,ab,ti) OR ((singl* NEAR/1 blind*):de,ab,ti) OR assign*:de,ab,ti OR allocat*:de,ab,ti OR volunteer*:de,ab,ti

#7 #3 AND #5 AND #6
Records: 1639

2 records selected

Question 8: In patients exhibiting distress resulting from and/or concomitant with their active cancer illness, is the use of non-pharmacological therapy, i.e. based on psychosocial and psychological interventions, indicated?
Question 9: In patients exhibiting depressive disorders resulting from and / or concomitant with their active cancer illness, is the use of non-pharmacological therapy, i.e. based on psychosocial and psychological interventions, indicated?

MEDLINE (PubMed) (1966 a December 2019)
((((((((((((("Neoplasms/psychology"[Mesh] OR "neoplasms/complications"[Mesh] OR neoplasms[Title/Abstract] OR cancer[Title/Abstract] OR neoplasm*[Title/Abstract] OR tumo*[Title/Abstract]))) AND (("Psychological Distress"[Mesh]) OR (distress[Title/Abstract] OR 'psychological distress'[Title/Abstract] OR 'emotional distress'[Title/Abstract])))))) OR (((((((((((("Mental Disorders/drug therapy"[Mesh] OR "Mental Disorders/epidemiology"[Mesh] OR "Mental Disorders/etiology"[Mesh])))) OR (((“Anxiety Neuroses”[Title/Abstract] OR “Affective Disorders”[Title/Abstract] OR “Neuroses Depressive”[Title/Abstract] OR “Neurosis Depressive”[Title/Abstract] OR “Endogenous Depression*”[Title/Abstract] OR “Depressive Syndrome*”[Title/Abstract] OR “Neurotic Depression*”[Title/Abstract] OR “Melancholia*”[Title/Abstract] OR “Unipolar Depression*”[Title/Abstract]))) OR ((((“psychiatric disorder”[Title/Abstract] OR “psychiatric disorders”[Title/Abstract] OR “psychiatric symptoms”[Title/Abstract] OR “adjustment disorder”[Title/Abstract] OR “adjustment disorders”[Title/Abstract] OR “stress-related disorder”[Title/Abstract] OR “stress-related disorders”[Title/Abstract] OR “major depression”[Title/Abstract] OR “depressive disorder”[Title/Abstract] OR “depressive disorders”[Title/Abstract])) OR (“mood disorder”[Title/Abstract] OR “mood disorders”[Title/Abstract] OR “demoralization”[Title/Abstract] OR “anxiety disorder”[Title/Abstract] OR “anxiety disorders”[Title/Abstract])) OR (“affective disorder”[Title/Abstract] OR “Neurotic Anxiety State”[Title/Abstract] OR “anxiety state”[Title/Abstract]))))))))) AND (((((("Social needs"[Title/Abstract] OR "psychosocial needs"[Title/Abstract] OR "unmet needs"[Title/Abstract] OR "supportive care needs"[Title/Abstract])) OR "Psychosocial health care needs"[Title/Abstract]) OR ("Health Services Needs and Demand"[Mesh] OR "needs assessment"[Mesh])) AND ((((((((((((("systematic review"[Title]) OR "meta-analysis "[Publication Type]) OR ("meta-analysis"[Title] OR "meta synthesis"[Title] OR "meta-analy*"[Title] OR "integrative review"[Title] OR "integrative research review"[Title])) OR "systematic literature review"[Title]) OR ("this systematic review"[Text Word] OR "pooling project"[Text Word]))) OR (("systematic review"[Title/Abstract]) AND review[Publication Type]))))))) OR ((((((((("Randomized Controlled Trial"[Publication Type]) OR "Clinical Trial"[Publication Type]) OR "drug therapy"[Subheading])) OR ((randomized[Title/Abstract] OR randomised[Title/Abstract] OR placebo[Title/Abstract] OR randomly[Title/Abstract] OR trial[Title/Abstract] OR groups)))) NOT ((("Animals"[Mesh]) NOT "Animals"[Mesh]) AND "Humans"[Mesh])))))
Records: 1673

Embase (Embase.com) (1974 a December 2019)
#1 'neoplasms psychology'/exp/mj
#2 neoplasms OR cancer OR neoplasm* OR tumo*:ti,ab
#3 #1 OR #2
#4 'depression'/exp/mj OR 'psychological distress' OR 'emotional distress' OR depression OR anxiety:ti,ab
#5 'psychosocial care'/exp/mj
#6 'psychosocial support' OR 'psychosocial support systems' OR 'psychosocial therapy' OR 'social therapy':ti,ab
#7 #5 OR #6
#8 #3 AND #4 AND #7
#9 'crossover procedure':de OR 'double-blind procedure':de OR 'randomized controlled trial':de OR 'single-blind procedure':de OR random*:de,ab,ti OR factorial*:de,ab,ti OR crossover*:de,ab,ti OR ((cross NEXT/1

over*):de,ab,ti) OR placebo*:de,ab,ti OR ((doubl* NEAR/1 blind*):de,ab,ti) OR ((singl* NEAR/1 blind*):de,ab,ti) OR assign*:de,ab,ti OR allocat*:de,ab,ti OR volunteer*:de,ab,ti
#10 #8 AND #9
Records: 174

2 records selected

Question 10: In patients exhibiting depressive disorders resulting from and / or concomitant with their active cancer illness, is the use of psychopharmacological therapy indicated?

MEDLINE (PubMed) (1966 a December 2019)
(((((((((((((("Neoplasms/complications"[Mesh] OR "Neoplasms/epidemiology"[Mesh] OR "Neoplasms/psychology"[Mesh])) OR (tumo*r[Title/Abstract] OR cancer[Title/Abstract] OR carcinoma[Title/Abstract] OR adenoma][Title/Abstract] OR adenocarcinoma[Title/Abstract] OR polyp[Title/Abstract] OR Neoplasia*[Title/Abstract] OR Neoplasm[Title/Abstract] OR "Malignant Neoplasms"[Title/Abstract] OR "Benign Neoplasms"[Title/Abstract]))))))))))))) AND ((((((((((((("Mental Disorders/drug therapy"[Mesh] OR "Mental Disorders/epidemiology"[Mesh] OR "Mental Disorders/etiology"[Mesh])))) OR ((("Anxiety Neuroses"[Title/Abstract] OR "Affective Disorders"[Title/Abstract] OR "Neuroses Depressive"[Title/Abstract] OR "Neurosis Depressive"[Title/Abstract] OR "Endogenous Depression*"[Title/Abstract] OR "Depressive Syndrome*"[Title/Abstract] OR "Neurotic Depression*"[Title/Abstract] OR "Melancholia*"[Title/Abstract] OR "Unipolar Depression*"[Title/Abstract]))) OR (((("psychiatric disorder"[Title/Abstract] OR "psychiatric disorders"[Title/Abstract] OR "psychiatric symptoms"[Title/Abstract] OR "adjustment disorder"[Title/Abstract] OR "adjustment disorders"[Title/Abstract] OR "stress-related disorder"[Title/Abstract] OR "stress-related disorders"[Title/Abstract] OR "major depression"[Title/Abstract] OR "depressive disorder"[Title/Abstract] OR "depressive disorders"[Title/Abstract])) OR ("mood disorder"[Title/Abstract] OR "mood disorders"[Title/Abstract] OR "demoralization"[Title/Abstract] OR "anxiety disorder"[Title/Abstract] OR "anxiety disorders"[Title/Abstract])) OR ("affective disorder"[Title/Abstract] OR "Neurotic Anxiety State"[Title/Abstract] OR "anxiety state"[Title/Abstract]))))))) AND (((((((("Psychotropic Drugs/adverse effects"[Mesh] OR "Psychotropic Drugs/pharmacokinetics"[Mesh] OR "Psychotropic Drugs/pharmacology"[Mesh])) OR ((Psychopharmaceuticals [Title/Abstract] OR "Psychoactive Agents"[Title/Abstract] OR "Psychoactive Drugs"[Title/Abstract] OR "Hallucinogens agents"[Title/Abstract] OR " tranquilizing agents"[Title/Abstract] OR " antipsychotics agents"[Title/Abstract] OR "anti-anxiety agents"[Title/Abstract])) OR ("psychotropic drug"[Title/Abstract] OR "psychotropic drugs"[Title/Abstract] OR antidepressant[Title/Abstract] OR antidepressants[Title/Abstract] OR "antidepressive agent"[Title/Abstract] OR "antidepressive agents"[Title/Abstract])))))))
Records: 449

Embase (Embase.com) (1974 a December 2019)
#1 'neoplasm'/exp/mj
#2 (tumo* OR canceror) AND carcinoma OR adenoma OR adenocarcinoma OR polyp OR neoplasia* OR neoplasm OR 'malignant neoplasms' OR 'benign neoplasms':ti,ab
#3 #1 OR #2
#4 'mental disorders psychology'/exp/mj
#5 'anxiety neuroses' OR 'affective disorders' OR 'neuroses depressive' OR 'neurosis depressive' OR 'endogenous depression*' OR 'depressive syndrome*' OR 'neurotic depression*' OR 'melancholia*' OR 'unipolar depression*' OR 'psychiatric disorder' OR 'psychiatric disorders' OR 'psychiatric symptoms' OR 'adjustment disorder' OR 'adjustment disorders' OR 'stress-related disorder' OR 'stress-related disorders' OR 'major depression' OR 'depressive disorder' OR 'depressive disorders' OR 'mood disorder' OR 'mood disorders' OR 'demoralization' OR 'anxiety disorder' OR 'anxiety disorders' OR 'affective disorder' OR 'neurotic anxiety state' OR 'anxiety state':ti,ab
#6 #4 OR #5
#7 'psychotropic agent'/exp/mj

#8 psychopharmaceuticals OR 'psychoactive agents' OR 'psychoactive drugs' OR 'hallucinogens agents' OR 'tranquilizing agents' OR 'antipsychotics agents' OR 'anti-anxiety agents' OR 'psychotropic drug' OR 'psychotropic drugs' OR antidepressant OR antidepressants OR 'antidepressive agent' OR 'antidepressive agents':ti,ab
#9 #7 OR #8
#10 #3 AND #6 AND #9
Records: 1031

<u>1 record selected</u>

Question 11: Should systematic screening for patient psychosocial needs be performed in cancer wards, with activation of a structured response strategy?

MEDLINE (PubMed) (1966 a December 2019)
((((((((((((((("Neoplasms/psychology"[Mesh]) OR ((neoplasms[Title/Abstract] OR cancer[Title/Abstract] OR neoplasm*[Title/Abstract] OR tumo*[Title/Abstract])))))) AND (((("Health Services Needs and Demand"[Mesh]) OR "Needs Assessment"[Mesh])) OR (Needs[Title/Abstract] OR 'Target Population'[Title/Abstract] OR 'Health Services Needs' [Title/Abstract] OR 'Needs Assessments'[Title/Abstract] OR 'Determination of Health Care Needs'[Title/Abstract] OR 'Assessment of Health Care Needs'[Title/Abstract])))) AND ((("management plans"[Title/Abstract] OR "management strategies"[Title/Abstract] OR "needs assessment"[Title/Abstract])) OR 'unmet supportive care needs'[Title/Abstract]))) AND ((((((((((((("systematic review"[Title]) OR "meta-analysis "[Publication Type]) OR ("meta-analysis"[Title] OR "meta synthesis"[Title] OR "meta-analy*"[Title] OR "integrative review"[Title] OR "integrative research review"[Title])) OR "systematic literature review"[Title]) OR ("this systematic review"[Text Word] OR "pooling project"[Text Word]))) OR (("systematic review"[Title/Abstract]) AND review[Publication Type]))))))) OR ((((((((("Randomized Controlled Trial"[Publication Type]) OR "Clinical Trial"[Publication Type]) OR "drug therapy"[Subheading])) OR ((randomized[Title/Abstract] OR randomised[Title/Abstract] OR placebo[Title/Abstract] OR randomly[Title/Abstract] OR trial[Title/Abstract] OR groups)))) NOT ((("Animals"[Mesh]) NOT "Animals"[Mesh]) AND "Humans"[Mesh]))))))
Records: 225

Embase (Embase.com) (1974 a December 2019)
#1 'neoplasm'/exp/mj
#2 (tumo* OR canceror) AND carcinoma OR adenoma OR adenocarcinoma OR polyp OR neoplasia* OR neoplasm OR 'malignant neoplasms' OR 'benign neoplasms':ti,ab
#3 #1 OR #2
#4 'health service'/exp/mj OR 'needs assessment'/exp/mj
#5 needs OR 'target population' OR 'health services needs' OR 'needs assessments' OR 'determination of health care needs' OR 'assessment of health care needs':ti,ab
#6 #4 OR #5
#7 #3 AND #6
#8 'management plans' OR 'management strategies' OR 'needs assessment' OR 'unmet supportive care needs':ti,ab
#9 #7 AND #8
#10 'crossover procedure':de OR 'double-blind procedure':de OR 'randomized controlled trial':de OR 'single-blind procedure':de OR random*:de,ab,ti OR factorial*:de,ab,ti OR crossover*:de,ab,ti OR ((cross NEXT/1 over*):de,ab,ti) OR placebo*:de,ab,ti OR ((doubl* NEAR/1 blind*):de,ab,ti) OR ((singl* NEAR/1 blind*):de,ab,ti) OR assign*:de,ab,ti OR allocat*:de,ab,ti OR volunteer*:de,ab,ti
#11 #9 AND #10
Records: 485

<u>2 records selected</u>

Question 12: For cancer patients of different linguistic and cultural backgrounds, can the presence of cultural brokers and/or interpreters improve patient-healthcare professional communication?
Question 13: Can cultural competence education for healthcare professionals improve communication with cancer patients of different linguistic and cultural backgrounds?

MEDLINE (PubMed) (1966 a December 2019)
(((((((((((((((("Neoplasms/complications"[Mesh] OR "Neoplasms/epidemiology"[Mesh] OR "Neoplasms/psychology"[Mesh])) OR (tumo*r[Title/Abstract] OR cancer[Title/Abstract] OR carcinoma[Title/Abstract] OR adenoma][Title/Abstract] OR adenocarcinoma[Title/Abstract] OR polyp[Title/Abstract] OR Neoplasia*[Title/Abstract] OR Neoplasm[Title/Abstract] OR “Malignant Neoplasms”[Title/Abstract] OR “Benign Neoplasms”[Title/Abstract])))))))))))))) AND ((((("Emigrants and Immigrants"[Mesh])) OR (("Cultural Diversity"[Mesh]) OR "Communication Barriers"[Mesh])))) OR (((migrant*[Title/Abstract] OR "culturally diverse"[Title/Abstract] OR "linguistically diverse"[Title/Abstract])) OR ("linguistic barriers"[Title/Abstract] OR "language barriers"[Title/Abstract] OR "social determinants of health"[Title/Abstract] OR "ratial disparities"[Title/Abstract] OR "health disparities"[Title/Abstract])))) AND ((("intercultural mediation"[Title/Abstract] OR "intercultural programs"[Title/Abstract] OR "culture broker"[Title/Abstract] OR "culture brokers"[Title/Abstract] OR "cultural broker"[Title/Abstract] OR "cultural brokers"[Title/Abstract] OR "culture brokerage"[Title/Abstract] OR "cultural brokerage"[Title/Abstract] OR culture mediat*[Title/Abstract] OR cultural mediat*[Title/Abstract] OR interpret*[Title/Abstract] OR translat*[Title/Abstract] OR "cultural competence"[Title/Abstract] OR "health-equity"[Title/Abstract] OR "health equity"[Title/Abstract]))
Records: 185

Embase (Embase.com) (1974 a December 2019)
#1 'neoplasm'/exp/mj
#2 (tumo* OR canceror) AND carcinoma OR adenoma OR adenocarcinoma OR polyp OR neoplasia* OR neoplasm OR 'malignant neoplasms' OR 'benign neoplasms':ti,ab
#3 #1 OR #2
#4 'migrant'/exp/mj OR 'cultural diversity'/exp/mj OR 'communication barrier'/exp/mj
#5 migrant* OR 'culturally diverse' OR 'linguistically diverse' OR 'linguistic barriers' OR 'language barriers' OR 'social determinants of health' OR 'ratial disparities' OR 'health disparities':ti,ab
#6 #4 OR #5
#7 #3 AND #6 AND ([cochrane review]/lim OR [systematic review]/lim OR [meta analysis]/lim OR [controlled clinical trial]/lim OR [randomized controlled trial]/lim)
Records: 193

2 records selected

Question 14: For families of advance cancer patients, are supportive psychosocial interventions indicated?

MEDLINE (PubMed) (1966 a December 2019)
(((((((((((((((((((("Neoplasms/complications"[Mesh] OR "Neoplasms/epidemiology"[Mesh] OR "Neoplasms/psychology"[Mesh])) OR (tumo*r[Title/Abstract] OR cancer[Title/Abstract] OR carcinoma[Title/Abstract] OR adenoma][Title/Abstract] OR adenocarcinoma[Title/Abstract] OR polyp[Title/Abstract] OR Neoplasia*[Title/Abstract] OR Neoplasm[Title/Abstract] OR “Malignant Neoplasms”[Title/Abstract])))) AND (((("Terminal Care"[Mesh]) OR "Terminally Ill"[Mesh])) OR ("End of life"[Title/Abstract] OR end-of-life[Title/Abstract] OR 'Terminal Care'[Title/Abstract] OR 'terminally ill'[Title/Abstract])))) AND ((((((((((((("Stress, Psychological/diagnosis"[Mesh] OR "Stress, Psychological/prevention and control"[Mesh]))) OR "Adaptation, Psychological"[Mesh])) OR (("Anxiety Disorders/diagnosis"[Mesh] OR "Anxiety Disorders/psychology"[Mesh])))) OR ("physiological support"[Title/Abstract] OR "psychosocial support"[Title/Abstract] OR "distress managment"[Title/Abstract] OR "physiological needs"[Title/Abstract] OR "psychosocial needs"[Title/Abstract] OR "physiological interventions"[Title/Abstract] OR "psychosocial interventions"[Title/Abstract]))))) OR ("Psychoeducational interventions"[Title/Abstract] OR "Psychoeducational servieces"[Title/Abstract]))))

Records: 626

Embase (Embase.com) (1974 a December 2019)
#1 'neoplasm'/exp/mj
#2 (tumo* OR canceror) AND carcinoma OR adenoma OR adenocarcinoma OR polyp OR neoplasia* OR neoplasm OR 'malignant neoplasms' OR 'benign neoplasms':ti,ab
#3 #1 OR #2
#4 'terminal care'/exp/mj OR 'terminally ill patient'/exp/mj OR 'end of life' OR 'terminal care' OR 'terminally ill':ti,ab
#5 #3 AND #4
#6 'mental stress'/exp/mj OR 'anxiety disorder'/exp/mj
#7 'physiological support' OR 'psychosocial support' OR 'distress managment' OR 'physiological needs' OR 'psychosocial needs' OR 'physiological interventions' OR 'psychosocial interventions' OR 'psychoeducational interventions' OR 'psychoeducational servieces':ti,ab
#8 #6 OR #7
#9 #5 AND #8
Records: 272

2 records selected
